# An online behavior change intervention to promote physical activity in adults with asthma: study protocol for a multicenter randomized controlled trial

**DOI:** 10.1186/s13063-022-06881-x

**Published:** 2022-12-07

**Authors:** Joice Mara de Oliveira, Manuela Karloh, Thiago Sousa Matias, Graziele Besen Barbosa, Patricia Duarte Freitas, Celso R. F. Carvalho, Karina Couto Furlanetto

**Affiliations:** 1 Graduate Program in Rehabilitation Sciences, Pitágoras-Unopar University (UNOPAR), 591 Marselha St., Londrina, PR 86041-14 Brazil; 2 Laboratory of Research in Respiratory Physiotherapy (LFIP), Department of Physiotherapy, State University of Londrina (UEL), 60 Robert Kock Ave., Londrina, PR 86038-350 Brazil; 3 Department of Physiotherapy, Center for Health Sciences and Sport, Santa Catarina State University (UDESC), 358 Paschoal Simone St., Florianópolis, SC 88080-700 Brazil; 4 Department of Physical Education, Scool of Sports, Graduate Program in Physical Education, Graduate Program in Public Health, Federal University of Santa Catarina (UFSC), Eng. Agronômico Andrei Cristian Ferreira, s/n - Trindade, Florianópolis, SC 88040-900 Brazil; 5 Department of Physical Therapy, School of Medicine, University of Sao Paulo (USP), 455 Dr Arnaldo Ave., São Paulo, SP 01246-903 Brazil

**Keywords:** Asthma, Behavior therapy, Physical activity, Sedentary behavior

## Abstract

**Background:**

Behavior change interventions have been the focus of recent studies, and the COVID-19 pandemic highlighted the importance of online interventions. However, no previous studies have investigated behavior change techniques to improve physical activity in adults with asthma through online intervention.

**Methods:**

This double-blind clinical trial will investigate the effectiveness of an online behavior change intervention in increasing physical activity and reducing sedentary behavior in adults with asthma, as well as in improving other clinical outcomes in short and medium terms. Patients with clinically stable moderate to severe asthma, who are physically inactive and do not have cardiovascular and/or osteoneuromuscular impairments will be randomized into control or intervention groups (23 in each). Both groups will carry out an online educational program (1 h). Additionally, the intervention group will receive weekly individual online sessions for 12 weeks of motivation-based behavior change intervention to promote an increase in physical activity and reduce sedentary behavior based on both self-determination theory and transtheoretical model. The intervention group will also receive an activity monitor with specific strategies related to it. Both groups will be reassessed immediately after the intervention and 6 months after that. The primary outcomes are physical activity and sedentary behavior, which will be objectively assessed by a triaxial accelerometer (Actigraph wGT3X-BT). Secondary outcomes are Asthma Control Questionnaire, Incremental Step Test, Sit-To-Stand, Timed Up-and-Go, 4-Metre Gait Speed, Asthma Quality of Life Questionnaire, Pittsburgh Sleep Quality Index, Epworth Sleepiness Scale, Actiwatch 2, and the Hospital Anxiety and Depression Scale.

**Discussion:**

The intervention is unprecedented and was carefully developed to joint most characteristics and techniques of both behavioral strategies (transtheoretical model and self-determination theory). Therefore, this intervention has the potential to improve physical activity levels and asthma management and reduce sedentary behavior. As a consequence, this novel intervention will improve global health in this population and support its use in clinical practice. The intervention will be carried out online with direct weekly contact with the therapist. Consequently, it has low implementation costs, might improve patient’s attendance, and has the potential to be largely offered elsewhere.

**Trial registration:**

ClinicalTrials.govNCT05241223. Registered on January 22, 2022.

## Background

Behavior change (BC) interventions have been used to promote physical activity (PA), including PA level increment [1]. Several BC models have been tested such as the self-determination theory (SDT). The SDT aims to facilitate BC by supporting individuals’ basic psychological needs (BPN) (i.e., autonomy, competence, and relatedness) and, consequently, fostering volitional and high-quality forms of motivation for behavioral engagement, including PA improvement or maintenance [2].

Evidence suggests that BC strategies focused on BPN are due to increase more internal forms of motivation and facilitate PA performance, PA maintenance, and positive changes in health outcomes [2, 3]. Relatedly, the transtheoretical model (TTM) observes that BC happens by stages (of change) and each stage represents the individuals’ motivational disposition to adopt/maintain or not a behavior. The TTM considers that BC involves a set of changing processes that people experience to progress through these stages, along with decisional balance, self-efficacy, and temptations [4].

Individuals with chronic diseases are more likely to engage in low physical activity levels [5]. In addition to the disease burden, low PA levels may result in even detrimental consequences, including high mortality risk [6]. In contrast, PA improves multiple health benefits, and recent literature has shown the importance of people’s pleasure and enjoyment in becoming more physically active [7]. Therefore, interventions using BC strategies are particularly important in this population [7].

Studies have suggested that regular PA is associated with a reduction of depression symptoms and systemic inflammation, as well as improvement in exercise capacity, asthma control, and sleep quality in adults with asthma [8, 9]. However, this population still performs less PA than people without asthma [10]. Few studies aiming at improving PA in adults with asthma through BC techniques have been developed [11–14]. They were effective in increasing PA; however, it remains unclear whether these results are maintained over time and whether an online intervention is also effective in people with asthma. Furthermore, none of these studies used the well-established SDT to facilitate BC.

## Methods

### Aims

The primary aim of this randomized clinical trial is to investigate the effectiveness of an online BC intervention based on both SDT and TTM in increasing PA and reducing sedentary behavior in adults with asthma. Secondary aims are (I) to observe the impact of this intervention on asthma control, functional capacity, quality of life, sleep quality, and symptoms of anxiety and depression; (II) to verify whether the BC intervention improves the satisfaction of BPNs, self-determination and autonomous forms of motivation for PA; and (III) to investigate if the effects of the BC intervention will be maintained in the medium term (6 months after the end of intervention).

### Design and setting

**Fig. 1:**
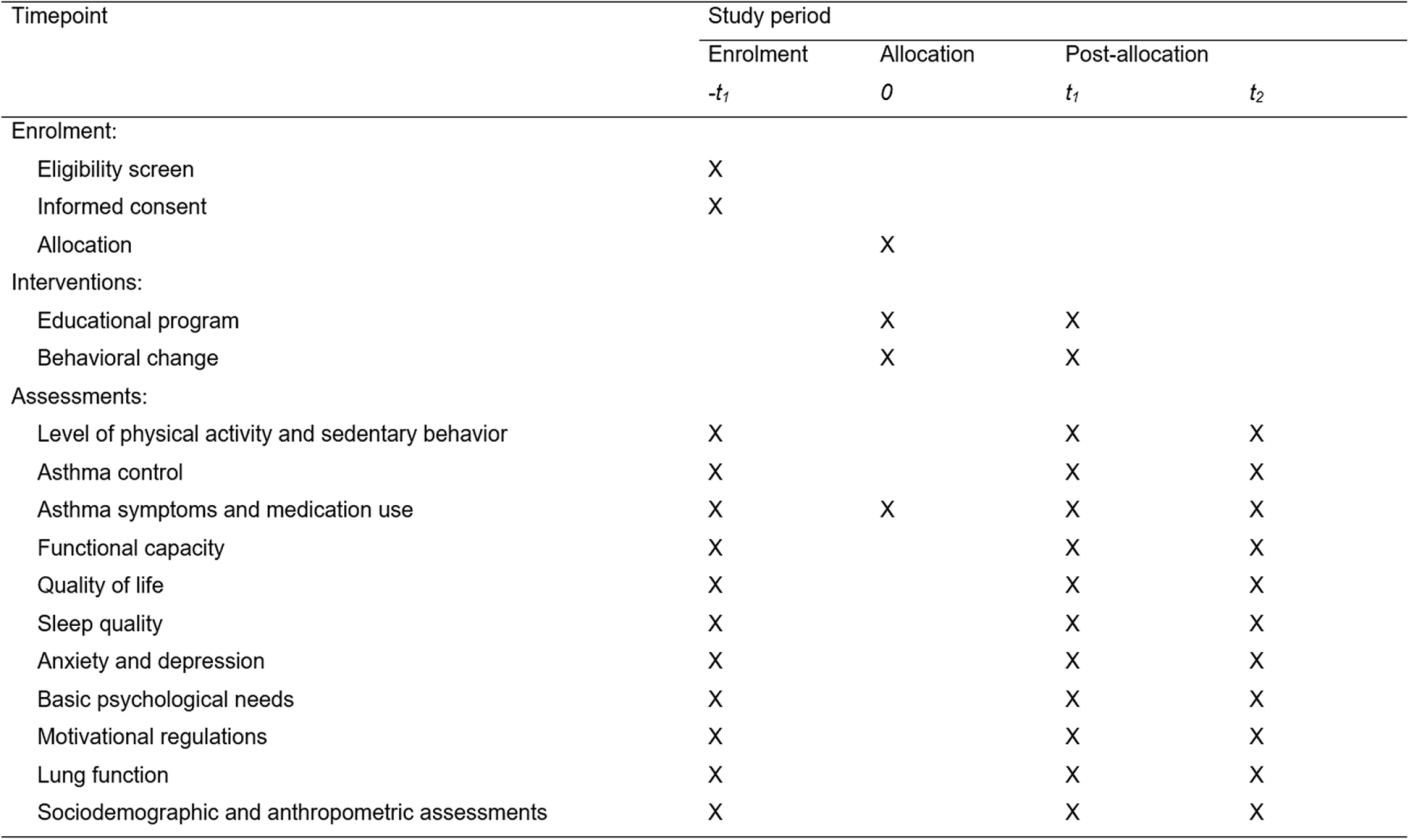
SPIRIT schedule of study enrolment, interventions, and assessments (*−t1=* enrolment; *0*= allocation; *t*_*1*_= post intervention; *t*_*2*_= 6 months after the end of intervention)

This double-blind (trial participants and outcome assessors) multicenter randomized clinical trial is expected to be carried out between February 2022 and November 2023. Patients will be contacted by a researcher involved in this study, informed about the study, and invited to participate. If they agree to participate, the inclusion criteria will be applied. All patients will be assessed before and immediately after the intervention (i.e., after the 12 weeks of intervention commencement), as well as 6 months after that (Fig. 1). Patients who discontinue the intervention protocol will be invited to a follow-up assessment. Research assessments will be carried out in person by trained assessors in both centers (Londrina and São Paulo), and the interventions will be carried out online by the main investigator from the coordinating center (Londrina).

**Fig. 2:**
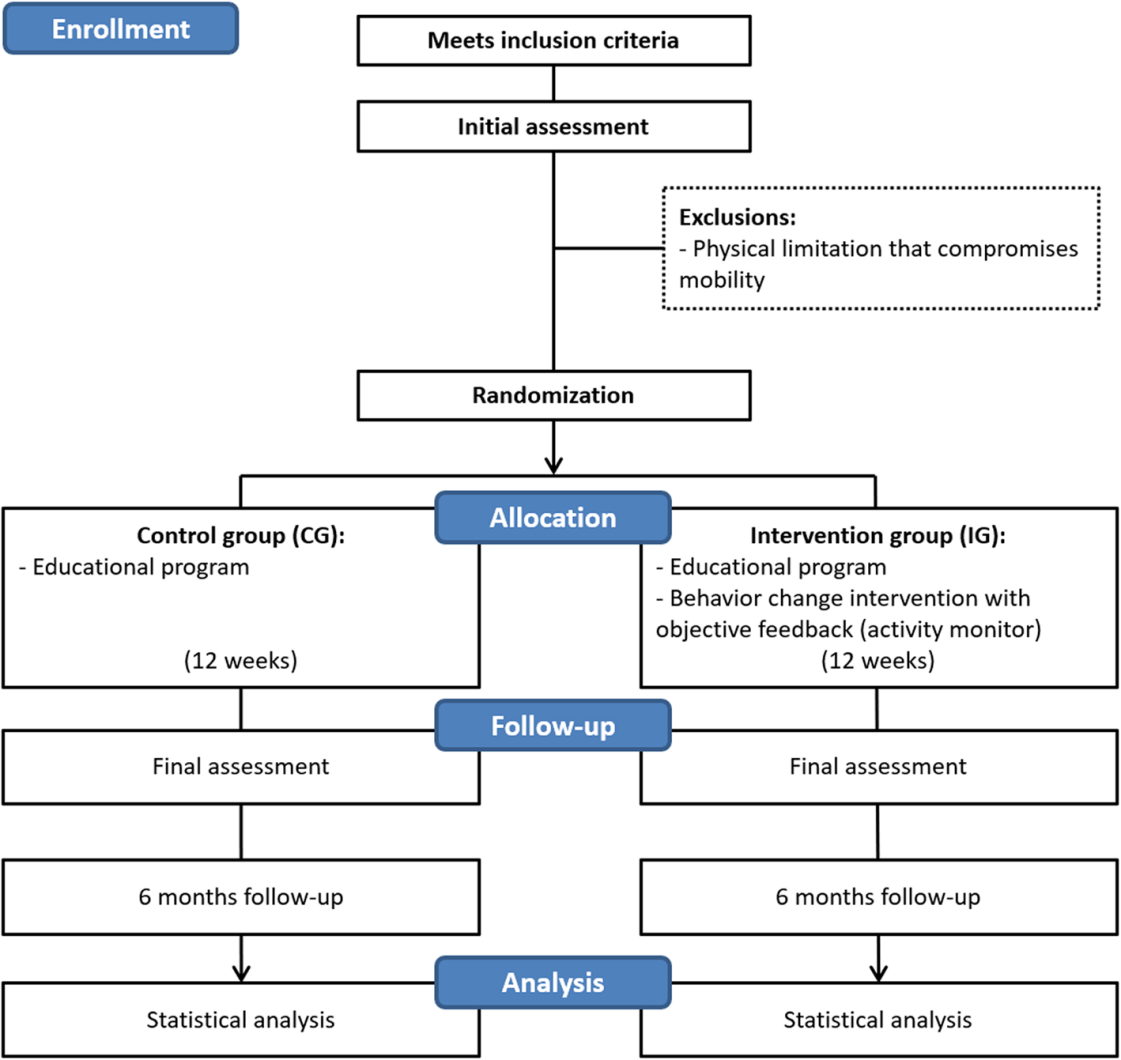
Study flowchart. The assessment will be carried out in one visit. Both groups will carry out a minimum educational program; the intervention group will receive weekly individual online behavior change intervention sessions for 12 weeks and will receive an activity monitor with its specific strategies. Both groups will be reassessed immediately after the intervention as well as 6 months after that

After the initial assessment, patients will be randomized into 2 groups: control group (CG) or intervention group (IG). Both groups will receive a minimal intervention (educational program), which will be explained further. The IG will receive weekly individual online sessions and also some online group sessions for 12 weeks of motivation-based BC intervention to promote PA and reduce sedentary behavior. The intervention group will also receive an activity monitor with instructions on how to use. Patients will be instructed to maintain their controller asthma medications during the intervention; however, if it is needed to change, they should record it in the asthma symptoms and medication control diary (explanation further). The study flowchart is shown in Fig. 2.

### Participants

Participants will be recruited through the referral of pulmonologists from outpatient clinics of Londrina and São Paulo, Brazil. Study publicization will be performed on social media too; however, patients who come in contact must be undergoing medical follow-up. This study will include people aged from 18 to 60 years, with a physician-based diagnosis of moderate to severe asthma (confirmed by checking the history of variable respiratory symptoms and treatment [15]) who underwent drug treatment for at least 6 months; with clinical stability for at least 1 month (without hospitalizations, emergency care use or medication changes). Additionally, absence of cardiovascular and/or osteoneuromuscular diseases that could interfere/hinder the performance of tests and physical activity, absence of lung diseases other than asthma, preserved cognitive function [16], non-smokers or ex-smokers with <10 pack-years, report being physically inactive in accordance with current physical activity guidelines [17], and people who are able to make video calls through any platform and/or free app available. The exclusion criteria comprise the presence of any physical limitation that compromises mobility and hinders PA performance during the participation period.

### Interventions

#### Educational program

The educational program will be delivered similarly to both groups through videos, images, and a booklet. After the initial assessment, all participants will receive a quick welcome video call, in which they will be randomized (see details further) and informed on how this program will work. After that, the educational videos and booklet will be sent daily for 5 subsequent days. Later, one educational image will be sent every other week up to the 12 weeks completion. Participants will be able to ask questions any time, if they want to. The program will cover information regarding the respiratory system, asthma pathophysiology and treatment, prevention strategies, and medication instructions [15]. It will also be briefly addressed the subjects: understanding PA and sedentarism, as well as the importance and benefits of being physically active and less sedentary [17].

#### Behavior change intervention

The IG group will receive an online intervention based on the TTM [4], along with SDT strategies [2]. A detailed protocol was carefully developed for the study aiming to make the most of the characteristics and techniques of each approach for the proper purposes. The intervention will take place weekly summing up 12 online individual sessions of approximately 30 min each. Four out of the 12 sessions will be carried out in group. The sessions will be made through a platform and/or application that makes video calls privately. Each session will address a topic (Table 1). In addition, in all sessions, the patients’ gain during the previous week will be recognized, as well as new goals will be set for the next week. The goals will always be individualized, realistic and achievable, based on the identification of each person's capacity and motivation [13].

**Table 1:** Behavioral change intervention sessions

Week	Theme	TTM	BPN	Main approach
1	Getting to know the patient		Autonomy	Test during the week types of PA which the patient can perform
			Competence	Train in the session PA/exercises that the patient can perform/knowing the activity monitor
			Relatedness	Know previous experiences of the patient related to PA/introduce the group
2	Why to be physically active?	Self-liberation (making a commitment)	Autonomy	Choose the when, how, and the type of PA that will perform
			Competence	Achievable goals, but with a certain challenge (contract)
			Relatedness	Contract is a commitment between the patient the therapist
3	Monitoring (group session) ^a^challenge	Contingency management (Using rewards)	Autonomy	Celebrating small victories
			Competence	Group challenge (gamification)
			Relatedness	Encouraging them to achieve the week group goals
4	Barriers to PA	Stimulus control (Managing the environment)	Autonomy	Recognition of the patient’s perspective on barriers
			Competence	Identifying barriers and indicating ways to overcome them
			Relatedness	Use an object with a color that represents the group
5	Sedentary lifestyle (group session)	Counterconditioning (using substitutes)	Autonomy	Recognizing environmental potential to reduce sedentary lifestyle and increase PA
			Competence	Focus goals on the process
			Relatedness	Encouraging reminders to reduce sedentary lifestyle and increase PA in the group
6	Goals review	Consciousness raising (getting the facts)	Autonomy	Visualizing the progress
			Competence	Controlling of patient evolution/learning calculation of average and goal of steps per day of the week
			Relatedness	Strengthening the bond between patient and therapist
7	Responsibility	Dramatic relief (paying attention to feelings)	Autonomy	Awareness of the importance of attendance
			Competence	Giving positive feedback
			Relatedness	Understanding feelings related to PA
8	Social support ^a^challenge	Helping relationships (getting support)	Autonomy	Discussion on what motivates to continue doing PA
			Competence	Challenge of doing PA with a person this week
			Relatedness	Searching for social support
9	Perceived benefits (group session)	Environmental revaluation and social liberation	Autonomy	Recognition of the patient’s perspective
			Competence	Feedback on how or not you achieved the result
			Relatedness	Exchanging experiences with the group
10	Possible relapses	Temptation	Autonomy	Action plan ideas
			Competence	Drawing up action plans for possible relapses
			Relatedness	Strengthen the group’s bond
11	Self-re-evaluation	Self-revaluation (creating a new self-image) and decisional balance (pros and cons)	Autonomy	Guidance with intrinsic objective
			Competence	Training/teaching things they failed to do
			Relatedness	Strengthen bonds with people close to them
12	Long-term goals (group session)		Autonomy	A leadership profile will be responsible for the group
			Competence	Providing encouragement and support
			Relatedness	Strengthen the group’s bond

The intervention will be delivered once a week for 12 weeks. The program will encompass the processes of change and other TTM techniques. Every session will include the BPN issues (autonomy, competence and relatedness) to facilitate intrinsic motivation from the self-determination theory. *TTM* transtheoretical model, *BPN* basic psychological needs.

^a^Challenges will be used as a form of gamification to move to “another level”

In addition, the IG participants will receive a Fitbit Zip (Fitbit, San Francisco, California, USA) [18] before the first session, which will be used to propose objective goals aimed at increasing PA. Patients will be instructed to wear it on the right side of the waist during the waking period and to remove it only for activities involving water and sleeping. Through the Fitbit Zip display, patients can visualize the number of steps given, as well as estimated energy expenditure and distance covered in the day. Patients will be advised to record these values daily, at the end of the day, in a diary delivered at the beginning of the intervention. This way, patients will be able to know how close they are to the goal and if they reached the goal.

**Fig. 3:**
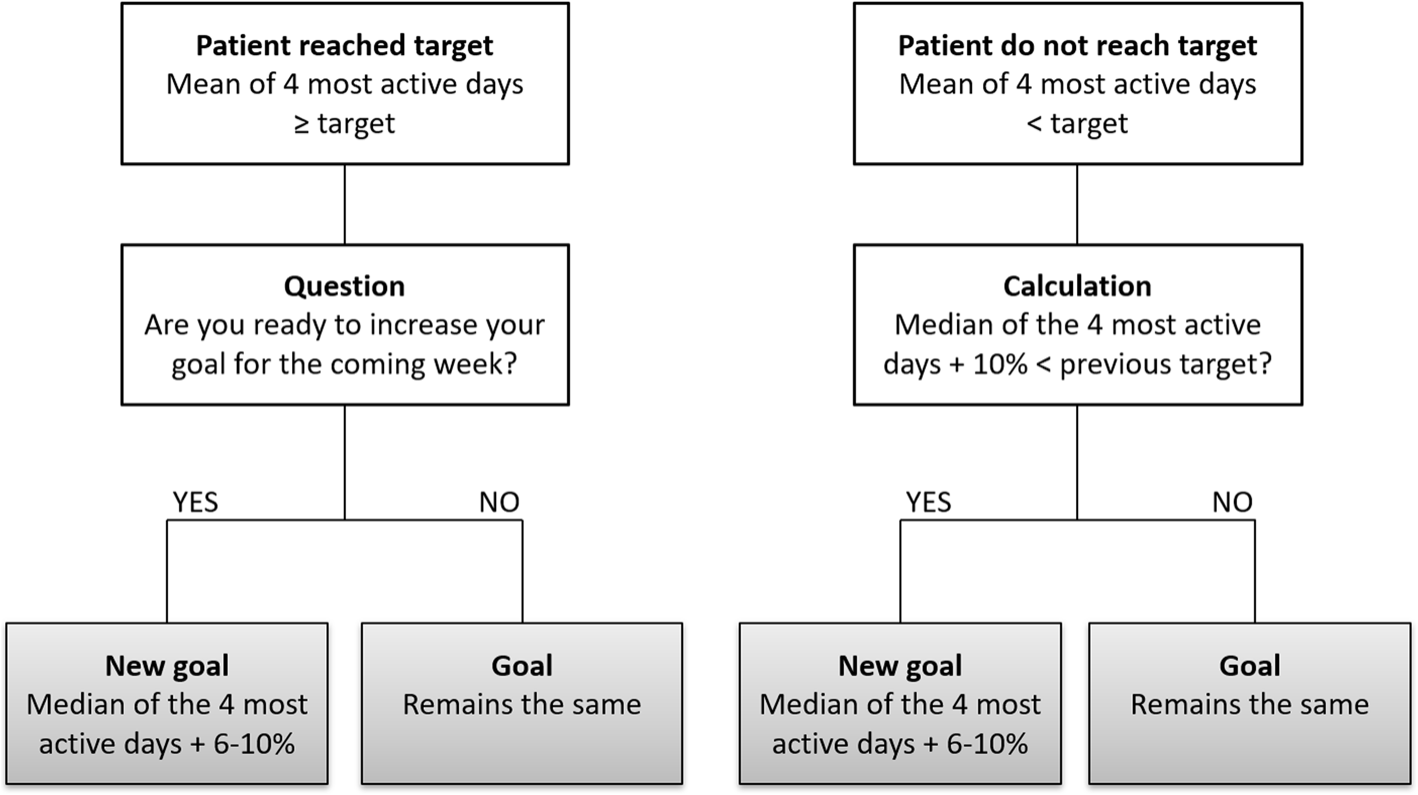
Flowchart of the weekly step goals progression for patients in the intervention group. This protocol was based on Demeyer et al.^19^

In the weekly sessions, the Fitbit Zip diary data will be revised, and the goal for the next week will be established from the average of the 4 most active days of the previous week [19] with an increase of 6 to 10% steps per week, if the patient feels ready for it (Fig. 3). If the patient does not reach the target in some week but performs an activity that makes him/her happy, he/she will be acknowledged for that. Additionally, a complex practice environment will be created around this device, in accordance with the SDT, such as the visualization of the number of steps evolution and competitions/games with other participants, among others.

### Outcome measures

#### Primary outcomes

##### Level of physical activity and sedentary behavior

It will be objectively measured by a triaxial accelerometer (Actigraph wGT3X-BT, Actigraph, Pensacola, FL, USA). The Actigraph wGT3X-BT will be initialized in a specific software (ActiLife v6.13.3) to collect data with a 60-hz sample rate. Participants will be instructed to wear the device on the waist (with an elastic belt) for 7 consecutive days. They should remove it only to perform water activities (i.e., showering) and sleep. This equipment provides as main variables the number of steps per day, time spent (in minutes and percentage of the day) in sedentary activities (<100 counts per minute), light-intensity PA (100–1951 counts per minute), and moderate to vigorous PA (≥1952 counts per minute) [20].

#### Secondary outcomes

##### Asthma control

The validated Asthma Control Questionnaire (ACQ) [21] on its 6-item version [22] will be applied. Participants must recall their symptoms during the previous week and answer the first 6 questions on a 7-point scale, ranging from 0 (no impairment) to 6 (maximum impairment) [23]. Items are equally weighted and the score is the average of all items.

##### Asthma symptoms and medication control diary

Patients will be given a paper diary in which they must record the asthma symptoms and exacerbations, as well as the use of medications for the disease during the 3 months of intervention [24]. Exacerbations will be defined as the use or increment of oral corticosteroids, for at least 3 days, due to worsening of symptoms; need for a doctor’s appointment or emergency care due to worsening of symptoms; or rescue bronchodilator use 4 times or more within 24 h for 2 consecutive days [25].

##### Functional capacity

Four different tests will be used: Incremental Step Test (IST), Sit-To-Stand (STS), Timed Up-and-Go (TUG) and 4-Metre Gait Speed (4MGS). All of them will be performed twice with a minimum rest period of 30 min and the best result will be used for analysis [26, 27]. To perform the IST, participants will be instructed to go up and down a step (20cm high, 40cm wide, 60cm deep). The cadence will be determined by an audio signal with an initial rhythm of 10 steps/min and will have increments of 1 step every 30 s until the participant’s tolerance. The result will be given both in the number of steps and in the vertical distance [27].

The STS will be performed in two different protocols: the 1-min (result in the number of repetition) and the 5-repetitions (result in seconds). Patients will be instructed to begin seated in a 46-cm high chair, with their arms crossed over the chest. With the command “go,” they will have to get up immediately and sit down repeatedly [26].

To perform the TUG, patients will be instructed to get up from a chair in the command “go,” walk in a straight line of 3 m on the floor, turn around, walk back to the chair and sit down. The time to perform the test at both normal and fast speeds will be counted [26].

In the 4MGS, the patients must walk a distance of 4 m marked on the floor, at the usual speed. The time, in seconds, needed to perform this activity is counted from the moment the patient starts to move until the moment the first foot completely crosses the 4-m line [26].

##### Quality of life

The validated Asthma Quality of Life Questionnaire (AQLQ) will be applied. Individuals should recall the frequency, intensity, and severity of health difficulties they have had due to asthma in the past 2 weeks. The total score is the emerging average of responses on 32 items on a seven-point scale, under four domains: activity limitations, symptoms, emotional function, and exposure to environmental stimuli. The score for each domain separately emerges from the average of their respective items [28].

##### Sleep assessment

Sleep quality will be assessed by the Pittsburgh Sleep Quality Index. This validated questionnaire consists of 19 questions regarding the last month, categorized into 7 components, classified between 0 and 3, with a total score ranging from 0 to 21 [29]. Daytime sleepiness will be assessed using the Epworth Sleepiness Scale, a questionnaire that assesses the probability of napping in 8 situations involving daytime activities. The total score ranges from 0 to 24 [30].

The Actiwatch 2 (Philips Respironics, Murrysville, Pennsylvania, USA) will be used to objectively measure sleep quality. This device must be used on the wrist of the non-dominant hand for 7 consecutive days, 24 h a day (the same period of use as the Actigraph, mentioned previously) [31]. During the days of use, patients must report in a diary information about bedtime, waking up, daytime nap time, and day/night-time symptoms. The following sleep variables will be measured: total time in bed, total sleep time, onset latency, sleep efficiency, wake after sleep onset, and the number of awakenings [31].

##### Anxiety and depression

Patients will answer to the Hospital Anxiety and Depression Scale [32]. This is a valid and simple 14-item questionnaire, with half of the items referring to anxiety and the other half to depression. Each item is rated on a 4-point scale, which gives a maximum subscale score of 21 for anxiety and depression, respectively [33].

##### Basic psychological needs

The Basic Psychological Needs in Exercise Scale will assess the participants’ perceptions of meeting basic psychological needs and exercise satisfaction. This validated scale consists of 12 items divided into three domains: autonomy, competence, and relatedness. The answers for each item range from 1 (I don’t agree) to 5 (I completely agree) [34].

##### Motivational regulations

The Behavioral Regulation in Exercise Questionnaire (BREQ-3) will be used to assess participants’ motivational regulations (amotivation, external, introjected, identified, integrated, and intrinsic regulation) to exercise/PA. This validated questionnaire consists of 23 items ranging from 0 (not true) to 4 (very true for me). The score is calculated for each motivational regulation as well as for the self-determination index [35].

##### Lung function

The assessment of lung function will be performed after the use of a bronchodilator using a spirometer (Pony FX COSMED, Italy). The technique will be performed according to the guidelines of the American Thoracic Society [36], determining the FEV_1_, forced vital capacity (FVC), and FEV_1_/FVC index. Reference values for the Brazilian population will be used [37].

##### Sociodemographic and anthropometric assessments

To characterize the sample, general data will be collected such as age, smoking habits, and personal history. Self-report of general comorbidities will be assessed by the validated Charlson index [38], and the most common comorbidities for people with asthma [39] will be questioned. Participants’ height and body weight will be objectively measured and the body mass index (BMI) will be obtained.

### Sample size calculation

The sample calculation was based on a previous study with adults with asthma in which two groups performed a 12-week intervention to increase physical activity, and the number of steps/day obtained in both groups (control and experimental) were normally distributed with the following values after the intervention: 6248±2030 and 8853±3320 steps/day, respectively [11]. Therefore, for the present study, a total sample of 38 people will be needed to reject the null hypothesis, with a power of 0.8 and a value of *P*<0.05. However, considering a 20% drop-out rate [13] throughout the study, the final sample size will be 46 people (23 in each group).

### Randomization and blinding

The block randomization sequence will be computer-generated and implemented by a person who will not participate in recruitment, evaluation, or treatment. The randomization sequence will be masked using opaque envelopes sealed and sequentially numbered. Each envelope will correspond to one of the two study groups and will be opened after the initial assessments through a video call. The nature of the intervention will not allow the blinding of the physiotherapist who will provide the behavioral intervention. However, the outcome assessors will be blinded to the allocation of patients into groups and patients will be masked to each treatment they are receiving.

### Statistical analysis

Statistical analysis will be performed after post-intervention and follow-up assessments using the software SPSS Statistics V22.0 (IBM, USA). Data distribution will be analyzed using the Shapiro-Wilk test. Variables will be presented as mean (SD), median [interquartile range 25–75%] according to data distribution. Categorical variables will be presented as absolute and relative frequency. The magnitude of statistical estimates will be presented using 95% CI. The results will be analyzed according to the intention-to-treat principle, as recommended by the CONSORT statement [40]. Per-protocol analysis might be performed to contrast results. Mixed model ANOVA will be used to identify the effect of the protocol to promote physical activity and reduce sedentary behavior. The repeated measures factor will be “time” (pre- and post-intervention evaluation) and the independent measures factor will be the “group” (control group or intervention group). To compare the proportion of patients that achieved the minimal clinical important difference in ACQ and AQLQ (0.5 points for both) [22, 41] between groups, the chi-square test will be used. Pearson or Spearman correlation coefficients will be used to verify the relationship between the changes in PA and changes in asthma control or quality of life. Values of *P* <0.05 will be considered statistically significant.

### Ethics

This study complies with the Declaration of Helsinki, was approved by Research Ethics Committee (5.005.112), and has been registered on ClinicalTrials.gov (NCT05241223). All results will be disseminated via article publication. Any changes made in this protocol will be reported in the research results article. All patients will sign the informed consent before participating and will receive one of its two copies. Data will be collected in paper forms and then entered in spreadsheets which will be double-checked. Participants will be assigned an identification code for all documentation; their personal data will be handled only by the research group and will not be disclosed. This study is not funded, therefore, there are no financial conflicts of interest.

## Discussion

BC intervention has been considered vital to optimize and maintain benefits from intervention in chronic care [42]. The intervention described in this study is unprecedented. It was carefully developed to joint most characteristics and techniques of both TTM and SDT [2, 4]. Additionally, it was included objective feedback to the patients (activity monitor) with weekly goals adapted from a previous study [19]. Furthermore, this intervention will be online, but with direct weekly contact with the therapist. Online interventions might improve participant attendance and has the potential to be largely offered elsewhere, including confinement conditions as experienced around the world with the COVID-19 pandemic. Studies in different populations showed that online intervention to increase PA is effective, easy to perform, and has low implementation costs. We hypothesize that this intervention will achieve positive results in the PA, sedentary behavior, asthma management, and global health of this population; therefore, supporting its use in clinical practice.

## Trial status

Protocol version: 1 (24/07/2022). Data recruitment started in February 2022 and is expected to finish in February 2023.

## Data Availability

Data sharing is not applicable to this article as no datasets were generated or analyzed during the current study.

## Abbreviations

4MGS: 4-Metre Gait Speed
ACQ: Asthma Control Questionnaire
AQLQ: Asthma Quality of Life Questionnaire
BC: Behavior change
BMI: Body mass index
BPN: Basic psychological needs
BREQ-3: Behavioral Regulation in Exercise Questionnaire
CG: Control group
FEV1: Forced expiratory volum in the first second
FVC: Forced vital capacity
IG: Intervention group
IST: Incremental Step Test
PA: Physical activity
SD: Standard deviation
SDT: Self-determination theory
STS: Sit-To-Stand
TTM: Transtheoretical model
TUG: Timed Up-and-Go
